# Patient and Clinician Perceptions of the Pulse Oximeter in a Remote Monitoring Setting for COVID-19: Qualitative Study

**DOI:** 10.2196/44540

**Published:** 2023-09-05

**Authors:** Andrea Torres-Robles, Karen Allison, Simon K Poon, Miranda Shaw, Owen Hutchings, Warwick J Britton, Andrew Wilson, Melissa Baysari

**Affiliations:** 1 Menzies Centre for Health Policy and Economics The University of Sydney Sydney Australia; 2 Sydney Local Health District Camperdown Sydney Australia; 3 School of Computer Science The University of Sydney Sydney Australia; 4 Royal Prince Alfred Virtual Hospital Sydney Local Health District Camperdown Sydney Australia; 5 Centenary Institute The University of Sydney Sydney Australia; 6 Faculty of Medicine and Health The University of Sydney Sydney Australia

**Keywords:** remote monitoring, patient experience, user experience, COVID-19, pulse oximetry, usability, acceptability, oximetry, wearable device

## Abstract

**Background:**

As a response to the COVID-19 pandemic, the Sydney Local Health District in New South Wales, Australia, launched the rpavirtual program, the first full-scale virtual hospital in Australia, to remotely monitor and follow up stable patients with COVID-19. As part of the intervention, a pulse oximeter wearable device was delivered to patients to monitor their oxygen saturation levels, a critical indicator of COVID-19 patient deterioration. Understanding users’ perceptions toward the device is fundamental to assessing its usability and acceptability and contributing to the effectiveness of the intervention, but no research to date has explored the user experience of the pulse oximeter for remote monitoring in this setting.

**Objective:**

This study aimed to explore the use, performance, and acceptability of the pulse oximeter by clinicians and patients in rpavirtual during COVID-19.

**Methods:**

Semistructured interviews and usability testing were conducted. Stable adult patients with COVID-19 (aged ≥18 years) who used the pulse oximeter and were monitored by rpavirtual, and rpavirtual clinicians monitoring these patients were interviewed. Clinicians could be nurses, doctors, or staff who were part of the team that assisted patients with the use of the pulse oximeter. Usability testing was conducted with patients who had the pulse oximeter when they were contacted. Interviews were coded using the Theoretical Framework of Acceptability. Usability testing was conducted using a think-aloud protocol. Data were collected until saturation was reached.

**Results:**

Twenty-one patients (average age 51, SD 13 years) and 15 clinicians (average age 41, SD 11 years) completed the interview. Eight patients (average age 51, SD 13 years) completed the usability testing. All participants liked the device and thought it was easy to use. They also had a good understanding of how to use the device and the device’s purpose. Patients’ age and device use–related characteristics (eg, the warmth of hands and hand steadiness) were identified by users as factors negatively impacting the accurate use of the pulse oximeter.

**Conclusions:**

Patients and clinicians had very positive perceptions of the pulse oximeter for COVID-19 remote monitoring, indicating high acceptability and usability of the device. However, factors that may impact the accuracy of the device should be considered when delivering interventions using the pulse oximeter for remote monitoring. Targeted instructions about the use of the device may be necessary for specific populations (eg, older people and patients unfamiliar with technology). Further research should focus on the integration of the pulse oximeter data into electronic medical records for real-time and secure patient monitoring.

## Introduction

Virtual care, including telehealth and telemedicine, has gained considerable momentum during COVID-19, as it allows patient care to be delivered remotely, reducing the risk of exposure to infection for health care professionals and patients [1-4]. Virtual models of care have been implemented for a range of chronic conditions, and clear benefits have been demonstrated, such as reductions in hospitalizations and emergency department visits [5].

A recent scoping review on the use of virtual care during COVID-19 noted the increased use of wearable health devices (eg, pulse oximeters and blood pressure monitors) as part of remote patient monitoring in these programs [6]. Wearable health devices have been used for health monitoring, the management of chronic diseases, and disease treatment [7]. These devices measure patients’ physiological parameters in real time, allowing health care providers to monitor a patient’s health condition from a distance and identify and prevent patient deterioration [8]. In the context of COVID-19, oxygen saturation (SpO_2_) is one of the critical indicators of patient deterioration [9], with low oxygen saturation shown to be linked to poorer clinical outcomes and higher mortality rates [9]. Similarly, monitoring of oxygen saturation can allow clinicians to identify when patients with mild COVID-19 are in need of hospitalization [10].

Understanding the experiences of users involved in the delivery (ie, health care professionals) and reception (ie, patients) of any health intervention is critical, particularly when the success of an intervention is dependent on its acceptability and uptake by end users [11]. A positive user experience has also been linked to better clinical outcomes and patient safety across different clinical conditions and settings [12]. Although patients’ perceptions of remote monitoring programs have previously been explored [13,14], we have limited evidence on end users’ experiences of wearable devices in virtual care, and in particular, the use of the pulse oximeter in this context. An in-depth evaluation of user perceptions toward the pulse oximeter is yet to be undertaken [15], and this research gap has been identified internationally [16].

Royal Prince Alfred Virtual Hospital (rpavirtual) was launched in February 2020 by Sydney Local Health District in New South Wales as the first full-scale virtual hospital in Australia [17]. Stable patients with COVID-19 who could be monitored and followed up remotely were admitted to the rpavirtual program and had 24-7 access to nursing and medical teams. Patients also received a wearable device (eg, a pulse oximeter) to monitor oxygen saturation levels [13,18].

Furthermore, while some wearable devices can transfer data to mobile apps, wearable device integration into electronic medical records (EMRs) has some challenges [19]. In the rpavirtual program, this is particularly problematic as clinicians must rely on patients to communicate their physiological measurements (eg, oxygen saturation levels) via other media (eg, over the phone). User-friendly wearable health devices that can be integrated into digital health infrastructure would be ideal for the development of real-time data transmission into EMRs. The evaluation of the user experience of wearable devices is the first step necessary for the development and ongoing refinement of these interventions [4].

The aim of this research was to explore the use, performance, and acceptability of the pulse oximeter by clinicians and patients in rpavirtual during COVID-19. We considered that this evaluation would allow us to identify necessary improvements to the service to support patient care and also provide broader insights on the integration of remote monitoring technology into a digital health infrastructure in other health care settings.

## Methods

### Study Design and Participants

This study used mixed methods and comprised 3 parts: an online survey (reported elsewhere), semistructured interviews, and usability testing with a think-aloud protocol. This study follows the Consolidated Criteria for Reporting Qualitative Research (COREQ) [20].

The first phase involved administering a user survey via the Research Electronic Data Capture (REDCap; Vanderbilt University) [21,22] database to rpavirtual patients and clinicians who had used the pulse oximeter. At the end of the survey, participants could express their interest in taking part in an interview (patients and clinicians) and usability testing (patients). Researchers then contacted these participants to provide further details about parts 2 and 3, answer any questions, and obtain informed consent.

To be included in this study, patients had to be 18 years or older, had to have been monitored by rpavirtual, and had to have used the pulse oximeter. Rpavirtual clinicians were eligible if they had monitored patients using the pulse oximeter for remote monitoring. Rpavirtual clinicians could be registered nurses, doctors, or staff from the digital patient navigator team, who assisted patients in the use of the remote wearables. To participate in the usability testing (part 3), patients also needed to have the pulse oximeter available at home. Interviews and usability testing were conducted between October 2021 and July 2022.

### Rpavirtual

COVID-19–positive and stable patients isolating at home or in hotel quarantine in the Sydney Local Health District since March 2020 were enrolled to the rpavirtual program. Patients received scheduled video or phone calls from rpavirtual clinicians to monitor patient symptoms and vital signs such as oxygen saturation with the pulse oximeter during the quarantine period. Wearable devices were delivered only to patients stratified as high risk [13].

### Pulse Oximeter

The iHealth AIR (manufactured for iHealth Labs Inc) wireless pulse oximeter, a consumer-grade wearable with a Bluetooth interface that could enable automatic data transfer into the EMR, was used for this study. The iHealth AIR has been approved by the Therapeutic Goods Administration in Australia, which has registered the iHealth AIR as a class IIa (low-moderate risk) product for intermittent pulse oximetry [23].

The iHealth AIR oximeter is powered by a rechargeable battery. After charging, the patient clamps the oximeter onto the ring, middle, or index finger of the nondominant hand and switches it on. The pulse rate and SpO_2_ appear on the oximeter’s built-in screen as values that can be relayed via Bluetooth to a smartphone or tablet device, on which an app stores the data [24].

As part of the rpavirtual program, patients used the device to communicate the readings when they were contacted by the rpavirtual clinician or anytime they wanted to check their oxygen saturation levels. For this study, only user experience with the pulse oximeter was evaluated. The Bluetooth interface was not tested.

### Interviews

Semistructured one-to-one interviews were conducted over the online platform Zoom (Zoom Technologies). Interview questions were informed by the Theoretical Framework of Acceptability (TFA) [11] and related to usability, task complexity, overall experience, and acceptance of the pulse oximeter in a remote monitoring setting. Interview questions were piloted with 2 users to ensure they flowed well and were well understood (the interview guide appears in Multimedia Appendix 1).

Interviews were conducted by ATR (a PhD-holding female postdoctoral fellow at the University of Sydney), who did not have any previous relationship with the participants. All interviews took place in English. For those patients who also participated in the usability testing, the testing was conducted immediately following the completion of the interview.

**Figure 1:**
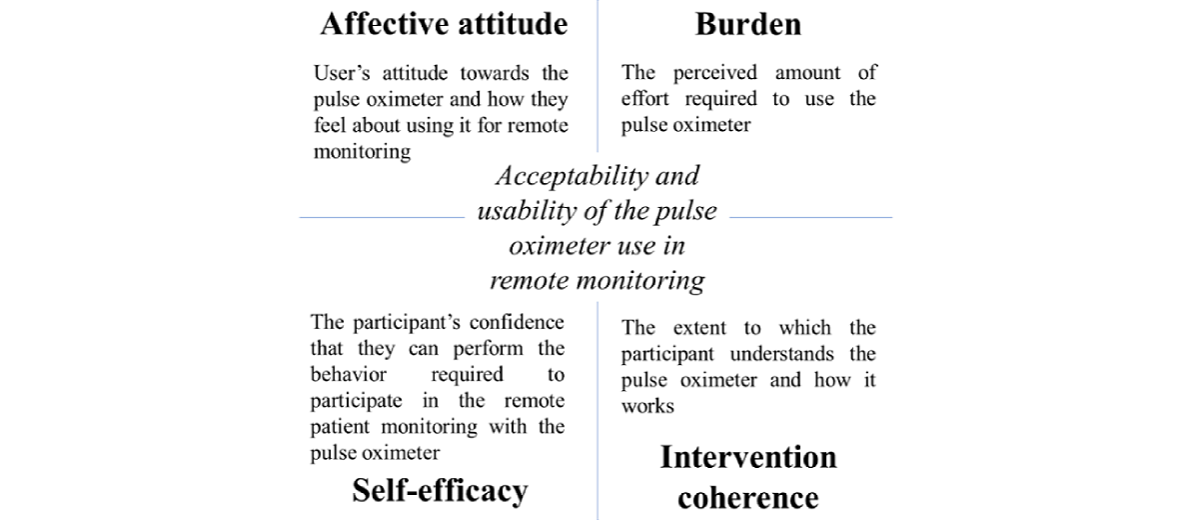
Constructs adapted from the Theoretical Framework of Acceptability.

Interviews were audio-recorded and transcribed by Otter (Otter.ai). Transcripts were reviewed by the researcher (ATR) to ensure they were accurate and nonidentifiable. Transcripts were then independently analyzed using a master code table generated in Microsoft Word (Microsoft Corp) by 3 researchers (ATR, KA, and MB). An inductive approach was used, but the analysis was guided by the TFA. That is, all themes identified in the data were mapped to the 4 constructs of the TFA: affective attitude, burden, intervention coherence, and self-efficacy constructs [11]. The definition of each construct is detailed in Figure 1.

The final 4-construct model resulted from a Delphi and a closed card sort process that allowed us to refine the conceptual model and test content validity based on overlapping items and existing literature on pulse oximeter user experience [25]. As a result, the constructs of affective attitude, perceived effectiveness, and ethicality were combined as the latter 2 constructs could be generalized to measure how the user feels about using the device. Similarly, the constructs of burden and opportunity costs were combined, as the amount of effort required by the user to use the device (ie, burden) could also include the extent to which patients and providers had to sacrifice benefits or comforts to use the device (ie, opportunity costs). This refined model was used to guide interviews.

Three researchers (MB, KA, and ATR) came together regularly to discuss identified themes. Inconsistencies in coding and mapping to the TFA were discussed between the coders until an agreement was reached. Interviews continued until thematic saturation was reached, and no new data were obtained from new participants. Saturation was discussed among the data analysis team following the coding of each new interview.

### Usability Testing

Usability testing was conducted via Zoom using a think-aloud protocol. The think-aloud method requires users to continuously verbalize their thoughts as they interact with the pulse oximeter, allowing participants’ thought processes, feedback, and emotional responses to be captured in real time [26]. This technique has been identified as one of the most effective ways to detect usability problems with digital health tools [27] and has been used in public health settings [28].

During usability testing, patients were asked (1) to use the pulse oximeter to generate a stable reading and (2) to charge the pulse oximeter while performing the tasks (and talking out loud). Usability testing sessions were video-recorded and the researcher (ATR) also noted down if the participant was able to successfully generate a reading on the device.

Video recordings were then reviewed by the interviewer (ATR) to identify any usability problems including problems encountered by users while trying to operate the device.

### Ethics Approval

Implied consent was obtained from participants who completed the user experience survey (ie, completing the survey constituted consent). An electronic consent form was sent to those participants interested in participating in an interview or usability testing. Electronic consent (e-consent) form information was stored in a password-protected REDCap database. Participants were reminded that their participation in the study was voluntary and they could withdraw at any point before deidentification. Ethical approval was granted by the Sydney Local Health District in July 2021 (X21-0182).

Participants were given an AUD $40 (US $55) online gift card after the interview was completed as reimbursement for their time.

## Results

### Overview

Thirty-five patients and 19 clinicians expressed their interest in participating in the qualitative study. All the participants were contacted, and 21 patients and 15 clinicians completed the interview. Interviews lasted on average 23 minutes, ranging between 12 minutes and 49 minutes for clinicians and between 12 minutes and 51 minutes for patients. Usability testing lasted on average 4 minutes (ranging between 1 minute 20 seconds and 10 minutes).

### Participant Demographics

The average patient age was 51 (SD 13) years, ranging from 27 to 80 years, and 17 (81%) were female. All patients had high school or a higher degree education, and 9 patients (43%) had a postgraduate degree. All patients had used the pulse oximeter at least twice per day and only 9 patients had used it for less than 5 days. More than half of the patients had never used a pulse oximeter before their experience with rpavirtual. Three patients described being referred to the hospital by the nurse while they were treated by rpavirtual due to low oxygen saturation levels. Two of the patients received oxygen treatment at a hospital emergency department.

The average clinician age was 41 (SD 11) years, ranging from 28 to 59 years, 9 clinicians (60%) were nursing staff, 5 (33%) were medical doctors, and 1 (7%) was part of the digital patient navigator team. Ten (67%) were female. Clinicians had been working for rpavirtual between 6 months and 1 year, and most were monitoring between 15 and 20 patients per day remotely.

The usability of the pulse oximeter was tested with 8 patients. Average age of patients was 51 (SD 11) years. Two patients (25%) had used a pulse oximeter before being enrolled in rpavirtual.

A summary of the positive and negative perceptions of the pulse oximeter that emerged from interviews can be found in Table 1.

**Table 1:** Summary of positive and negative patients and clinicians’ perceptions about the use of the pulse oximeter in remote monitoring.

	Positive perceptions	Negative perceptions
Affective attitude	Liked the device The device allowed monitoring at home The device helped to detect early deterioration	Risks associated with the use of the PO^a^: user dependent and anxiety due to inaccurate readings
Burden	Easy to use: put on, get a reading, and charge	Factors negatively impacting accurate use (eg, unsteady hand and cold fingers)
Intervention coherence	Good understanding of device purpose	N/A^b^
Self-efficacy	Sufficient training for patients about the use of the PO Good knowledge about the pulse oximeter (eg, general use, how to charge, and thresholds) Previous experience using the PO	N/A

^a^PO: pulse oximeter.

^b^N/A: not available.

### Affective Attitude

Overall, participants mentioned they would recommend remote monitoring with the pulse oximeter to their families or friends. Patients said they would recommend it as a tool to monitor their symptoms at home safely during COVID-19:

It was successful.... For my case, I didn’t have to go to hospital. And I think that’s exactly the right way to treat people who have COVID-19. Unless they need to be at hospital they should be isolating at home.

Patient #5

Most clinicians viewed the pulse oximeter as essential for preventing people from going to the hospital and for monitoring patients at home when they are isolating with COVID-19:

Oh, 100%. Yeah, definitely. It makes life so much easier. In terms of virtual health, it’s telemedicine. It’s great. I think it’ll be a game changer. Definitely, because at rpavirtual we’re working at a lot of other models of care to try to keep patients at home without having to send them into hospital and having a wearable device like that, that has that capacity to you know, remotely assess them makes life so much easier.

Clinician #4

This was consistent with patients’ views, as many patients mentioned that the device allowed them to stay home and avoid going to the hospital while having COVID-19.

A key benefit of the pulse oximeter reported by clinicians was the early detection of patient deterioration so that the patient could get treatment quickly. A clinician mentioned: “I think it definitely allowed us to pick up clinical deterioration earlier in some patients” [Clinician #7]. The device was also perceived to act as a safety net:

But having that sort of security of the pulse oximeter there gives me a bit more freedom and a bit more, I guess, confidence that I can manage this patient remotely rather than having to send them to the emergency department straightaway.

Clinician #15

Some patients explained that the pulse oximeter provided “peace of mind,” reduced anxiety, and gave them control of their clinical condition during COVID-19. For example, a patient said,

At the time when I got COVID I was pretty unwell, I had delta…I know, people’s saturations, oxygen saturation can deteriorate at certain times of the infectious period. So having that as a feedback mechanism, I think, reduced kind of a bit of anxiety.

Patient #12

Some patients and clinicians mentioned that the device was not suitable for everyone. Older patients, children, and people not familiar with the technology were more likely to struggle using the device. A small number of clinicians reported patients becoming obsessed with taking a reading:

Sometimes they may be using the pulse oximeter because sometimes people get really anxious and they use the pulse oximeter like 10, 20 times in a day, checking every minute,... and sometimes they get a wrong reading, and they panic.

Clinician #2

Both user groups also indicated that the device readings were not always accurate, and this inaccuracy could lead people to become more obsessed with taking a reading and could lead to anxiety and frustration.

### Burden

Overall, both patients and clinicians reported that the pulse oximeter was easy to use (eg, put on, turn on, charge, and read the value). Some patients also mentioned that it was easy to learn how to use the pulse oximeter. One patient said:

It’s very simple and easy to use. It’s very simple, it’s just straightforward. I don’t know. It comes with a cable, put it into a charger, the USB charger. And then charge it. And then turn it on. It was very easy. It’s very self-explanatory. There are not very many steps to it…. To see the value, it was very clear.

Patient #15

And a clinician explained:

I think it’s pretty easy. I think most patients that I’ve talked to seemed to have a good handle on it...you just have to put it on your finger and turn it on, and I think most people manage that.

Clinician #5

Participants described several factors that negatively impacted the accurate use of the pulse oximeter. In addition to the user factors described above, clinicians and patients explained that accuracy was impacted by finger position, keeping a steady hand, keeping warm hands or fingers, and nail polish. Users also said they had to wait to get a good reading, and the device needed to be charged to work properly. For example, one clinician said: “If you use it when it’s too cold or didn’t wait for about 30 seconds before it can get calibrated, the result is invalid” (Clinician #1).

Clinicians said that the pulse oximeter was more difficult to use for older patients:

Patients who seem to have trouble with, generally elderly, who weren’t comfortable with devices, generally, just with the concept of how to plug it in and charge it, turn it on. A lot of elderly people are completely fine with it. But I think, you know, for the people who weren’t, there definitely was apparent that they’re more likely to be older.

Clinician #7

Some clinicians and patients also reported difficulties with charging or checking the battery level of the pulse oximeter:

There’s no way for me to monitor whether the charge is going up. It just has a blinking green light. It doesn’t say to you: I’m finished.

Patient #17

It was just difficult to figure out which way around to put the charger.

Patient #4

### Intervention Coherence

Almost all the participants seemed to have a good understanding of the purpose of the pulse oximeter, how to use the device, and what values indicated poor oxygen saturation (eg, knowledge of the thresholds). A clinician said:

So main purpose, obviously, is to measure the oxygen saturations in the blood, to monitor patients... to make sure that they’re stable, and to make sure that their respiratory function is still being maintained. And...if it does drop then obviously we need to escalate.

Clinician #15

One patient said:

My understanding is that it measures the concentration of oxygen in my blood which indicates how well my lungs are getting oxygen into my bloodstream which I understand the COVID-19 interrupts and then it checks at the end.

Patient #7

Some clinicians were also able to explain the technical aspects of how the pulse oximeter works (eg, it sends light through the digit and measures the oxygen, it uses infrared light that is absorbed by proteins, and measures the light reflected to give a percentage of oxygen and it detects how much of the hemoglobin is oxygenated).

### Self-Efficacy

Most patients reported that they had received sufficient training and support from the rpavirtual staff on how to operate the pulse oximeter. For example,

They gave me the box with the instructions. And they explained on the phone how to use it. I mean, it’s pretty easy you stick it on your finger and you read it out.

Patient #2

When asked to describe the process of using the device, most patients correctly described how to use the pulse oximeter and how to communicate the reading to the rpavirtual team:

So, I put it on. Well obviously, when you put it on, you need to make sure that the green side is up upon your finger. So, you can actually...read it. And then give it a few moments, a few minutes, few seconds to stabilize and soon you get a reading. And if the reading is not right then...take some deep breaths and try and get a little reading to come up.

Patient #11

Both patients and clinicians appeared to know what to do, or troubleshoot, if the device was not giving a reading or if the reading was too low. This included warming the hands, taking deep breaths, repeating the reading, and keeping the hand steady. One clinician said:

So, you get them to swap the finger, the hands might be cold. So, you know, sometimes we get them to warm up their hands. And then sometimes I just give it five minutes.

Clinician #15

### Findings From Usability Testing

Key results from the usability testing appear in Textbox 1. Patients were highly consistent in the steps taken to obtain a reading using the pulse oximeter, as shown in Textbox 1. Only 1 patient deviated from this process and pressed the Start button first and then placed their finger into the pulse oximeter (ie, reversed steps 2 and 3). Most of the patients used the pulse oximeter on their left hand and index finger.

**Usability testing results.**

**Steps taken to obtain a reading using the pulse oximeter**

- Open the clamp of the pulse oximeter
- Place the middle, ring, or index finger into the rubber opening (most did this nail-side up)
- Press the Start button
- Keep your hand still
- Read the measurement displayed on the device
- Remove the device

Number of patients who completed the task successfully: 7/8

Out of 8 patients, 7 used the pulse oximeter and generated a reading on their first attempt. One patient had to try several times in order to generate a reading and this caused the patient to become frustrated:

I have always been frustrated with this oximeter because I wanted it just to give me a good reading even if it is 91...The oximeter has been very frustrating. This one doesn’t seem to work well. I stick my finger right to the end. We have always been unhappy with it. It doesn’t work on my finger on my right hand.... My hand is not cold actually. This oximeter is all over the place. I don’t rely on this very much any longer.

Most of the patients said that the pulse oximeter was easy to use or described the process while thinking out loud. For example, 1 patient said:

That’s all you have to do, if you just turn it on and put it on your finger and that’s it. Just put it on my finger and then power on. It is easy. You don’t have to think about it. It works automatically. Just put it on, and then press the power button. Give it a few moments because it is trying to get the reading and then it is done. That’s it.

All patients that took part in usability testing knew how to charge the device. A patient mentioned: “I just plug it to the charging connecter there (in the device). It is a standard USB charging port. It was easy.” Other patients said,

You can see where to put the little attachment where to plug it in. I opened it up, that is how easy. And it is a universal kind of USB, so if I lost that cord, I can use other type of USB cord. It is quite simple to use.

## Discussion

### Principal Results

This study revealed that the pulse oximeter was highly acceptable to both clinicians and patients, with both groups expressing more positive perceptions than negative perceptions about the device. Both clinicians and patients identified several benefits of remote monitoring using the pulse oximeter, reported that the device was easy to use, and had a good understanding of its purpose. The main negative views were related to the device not being suitable for everyone (eg, patients unfamiliar with technology), and the device’s accuracy being dependent on exactly how the device was used (eg, warmth of hands).

Participants of this study mentioned the pulse oximeter was beneficial in preventing patients from going to the hospital, allowing them to stay at home instead while recovering from COVID-19. Patients’ preferences to remain at home could be because of a fear of going to the hospital during the pandemic, a desire to leave hospital space for people who may need it, or being able to work at home [14]. Avoiding unnecessary hospital admission while allowing patients to be remotely monitored may protect patients and the community from exposure to infection and reduce the burden on the health care system [1]. Patients may also feel more comfortable being in a familiar home environment while recovering from COVID-19 if they are still being monitored by health care professionals. A user-friendly device and available clinical remote support may contribute to patients feeling more confident in monitoring their health.

The pulse oximeter was seen to be easy to use, including putting the pulse oximeter on and obtaining a reading, and both patients and providers understood its purpose. Different models of technology acceptance have linked the perceived ease of use to user acceptance and perceived usefulness [29]. Our result likely reflects the level of training and instructions provided to end users. All participants commented that printed instructions delivered with the pulse oximeter seemed to be clear and sufficient to patients, and some patients felt they did not need to call rpavirtual for further training. An initial call with a nurse was seen as useful and sufficient for learning how to use the device and understand the readings, and participants also reported that they could contact the remote monitoring team anytime. This may have played an important role in patients correctly using the device and engaging in the intervention by providing the knowledge and skills required to perform the task (ie, using the pulse oximeter). The training was also clearly effective in communicating the device’s purpose, with most participants demonstrating a good understanding of the purpose of the device and how to use the device correctly. Patient education and training on self-using the pulse oximeter has been identified as one of the recommendations when setting up and evaluating outcomes in remote monitoring interventions to ensure that patients’ measurements are accurate, and the intervention is effective [30]. The findings of this study indicate that patient training and education should also highlight factors impacting the accuracy of the reading, as this is likely to reduce anxiety or frustration with the pulse oximeter.

Our results suggest that instructions should be targeted to specific patient education needs as not all patients may experience the same challenges when using the pulse oximeter. Addressing the specific needs of user groups such as older people or people unfamiliar with technology would be necessary to ensure the device is acceptable and accessible to all user types. Factors such as age and digital literacy skills may influence digital health literacy [31], and therefore may also influence the adoption of new technologies and digital interventions in these groups of patients. Increasing health information and digital knowledge (eg, accurate use of the device) can reduce this gap [32]. In moving forward, additional or customizable education and information for these groups may improve usability and acceptability.

Psychological impact was also described by patients and clinicians. Obsession with taking a reading should also be considered when using the pulse oximeter remotely. Becoming fixated on self-monitoring has been identified as an unintended consequence of the use of wearables in health care [33]. Not generating a “good” reading appeared to lead patients to engage in the behavior (eg, taking a reading) repeatedly until a “good” reading was achieved. Inaccuracy of the pulse oximeter readings leading to anxiety and frustration should also be considered. Although the use of wearable technologies has many benefits, for some patients, it has been linked to psychological distress such as anxiety due to inaccurate data or readings that could be misinterpreted [34], with anxiety being a factor impacting technology acceptance [35].

### Comparison With Prior Work

The benefits of the pulse oximeter, as reported in this study, such as early detection of patient deterioration, have been highlighted in previous research. Signs of COVID-19–positive patient deterioration, for example, a higher risk of hospitalization and respiratory distress, have been associated with lower oxygen saturation levels [10]. Early identification of signs of patient deterioration (eg, declining or low oxygen saturation) might allow a quicker response to the patient’s clinical health condition. Also, a prompt escalation of patients who may clinically deteriorate for further assessment and treatment may prevent adverse patient outcomes (eg, worsening to severe COVID-19 disease and an extended hospital stay). This was reflected in our study where a small group of patients received prompt treatment and assessment after they were referred by nurses to the hospital due to low SpO_2_ levels while being remotely monitored. Detecting early variations in SpO_2_ levels might also be important in patients experiencing hypoxemia without showing any signs of respiratory distress (eg, “happy” hypoxemia), as rapid deterioration can occur if oxygen levels drop [36].

In line with previous research [37,38], clinicians identified a range of factors that affected the use of the pulse oximeter. Factors such as the finger position, avoiding nail polish, waiting time to get a reading, warming up the fingers, or keeping the hand steady while getting a reading may impact on the use of the pulse oximeter in remote monitoring and, therefore, in the acceptability of the intervention. To ensure these factors do not impact accurate use, instructions about the use of the device should be reviewed to include these factors to not only educate patients but also train clinicians delivering the intervention. These results support the recommendation of including patient education and training on self-using the device for remote monitoring to ensure the effectiveness of the intervention [30]. Moreover, the interaction between the clinician and the patient when providing education has resulted in the improvement of patient’s health outcomes [39]. Other factors like having darker skin may need to be considered when using the pulse oximeter in remote monitoring, as there is growing evidence of a decline in device accuracy in such patients [40]. Ease of use of the pulse oximeter was mentioned by patients in a study evaluating patients’ perspectives on a home monitoring service during COVID-19 [14]. Positive views toward the support provided by the clinicians and benefits of the pulse oximeter such as allowing patients to avoid hospitalization had also been reported in similar studies of remote monitoring [14,41], reinforcing the importance of clinical support in remote monitoring interventions.

### Limitations

There were some limitations in this study. The recruitment of participants was limited to those who expressed interest in the qualitative study when completing a user experience survey, and as such, our findings may not reflect the views of all users. We continued to recruit participants until thematic saturation was reached; however, we acknowledge that not all patient populations were represented in our sample (eg, dark-skinned individuals and individuals with health conditions impacting on the use of the device such as arthritis).

Our findings are limited to the rpavirtual intervention using the pulse oximeter for remote monitoring during COVID-19 and may not reflect the experiences of users in other settings. However, some of the perceptions reported in this study may be used for the design and improvement of similar interventions.

### Conclusions

The findings of this study revealed that the pulse oximeter in remote monitoring during COVID-19 was highly acceptable to most patients and clinicians, and easy to use. However, the device may not be suitable for all patient cohorts, and if required to be used by older patients, those unfamiliar with technology, or those with specific conditions (ie, poor circulation and arthritis) the device should be accompanied by additional education and instructions, particularly information related to factors that may impact on accurate readings being obtained with the device (eg, finger position, steady hand, and device charging process).

Due to its high user acceptability and its Bluetooth connectivity, the pulse oximeter evaluated in this study could be suitable for broader implementation at RPA Virtual hospital and other virtual care settings including for the real-time transmission of physiological measurements such as oxygen saturation levels from the pulse oximeter to the EMRs. This integration could aid clinical management in a timely and secure manner and improve patient care.

## Appendix

Multimedia Appendix 1 Interview guide.

## Abbreviations

COREQ: Consolidated Criteria for Reporting Qualitative Research
EMR: electronic medical record
REDCap: Research Electronic Data Capture
SpO2: oxygen saturation
TFA: Theoretical Framework of Acceptability

## Data Availability

The data sets generated and analyzed during this study are not publicly available due to privacy or ethical restrictions but are available from the corresponding author on reasonable request.
