# Supplementary material for: Patient and Clinician Perceptions of the Pulse Oximeter in a Remote Monitoring Setting for COVID-19: Qualitative Study
Source: J Med Internet Res. 2023 Sep 5;25:e44540. doi: 10.2196/44540 (PMC10482056; doi:10.2196/44540)
Supplement: Multimedia Appendix 1 [file jmir_v25i1e44540_app1.docx]

**APPENDIX A – INTERVIEW GUIDE**

Interview questions for patients:

1. Can you start off by telling me roughly how many times you’ve used the pulse oximeter and your overall impression of the device? Did you get it since the first day you were at rpavirtual? Have you used a similar device before?

2. Do you know why you were given a pulse oximeter to use? What is your understanding of what the pulse oximeter does? How does it work? What is its main purpose?

3. What are the main benefits of using the pulse oximeter at home or in the hotel?

4. Can you think of any risks with using the pulse oximeter at home or in the hotel?

5. When you were given the pulse oximeter, did you get any training on how to use it, or get any instructions from the rpavirtual staff? What did you think of this training and/or support? If you had ongoing problems using the device, was there support available? Do you feel like you had enough support to use the device?

6. How easy is the pulse oximeter to use? Have you had any problems using the device? If yes, can you describe these?

7. Does the device work all the time? Have you encountered any problems with getting a reading? Can you describe these? How did you feel?

8. How easy is it to read the value on the device? How do you use the information that comes out of the device?

9. How do you communicate the pulse oximeter readings to the rpavirtual team? Have you had any problems with this step?

10. Would you recommend remote monitoring with the pulse oximeter to your friends? Why or why not?

11. Can you think of any ways that remote monitoring with the pulse oximeter could be improved?

12. Do you have any other comments you’d like to make about the pulse oximeter or your remote monitoring experience in general?

Interview questions for clinicians

1. Can you start off by telling us roughly how many times your patients have used the pulse oximeter with you as part of their clinical care? What is your overall impression of the device?

2. Do you know what the pulse oximeter does and how it works? What is its main purpose?

3. What are the main benefits of patients using the pulse oximeter at home or in the hotel?

4. Can you think of any risks with patients using the pulse oximeter at home or in the hotel?

5. When the pulse oximeter was introduced, were you given any information or training on how it operates from within the rpavirtual team or ICT Services? Was this useful?

6. What about ongoing support, if your patients have problems using the device at home? Is there enough support and is it helpful?

7. How easy do you think the pulse oximeter is for patients to use? Have you had any problems using the device with your patients? If yes, can you describe these?

8. What about with understanding the readings, is this easy to do?

9. How do you use the information that comes out of the device?

10. How does your patient communicate the pulse oximeter readings to you or the rpavirtual team? Have you had any problems with this step?

11. Would you recommend remote monitoring with the pulse oximeter to your colleagues? Why or why not?

12. Can you think of any ways that remote monitoring with the pulse oximeter could be improved?

13. Do you have any other comments you’d like to make about the pulse oximeter or your remote monitoring experience in general?
